# Quality of Life after Flatfoot Surgery in the Pediatric Population

**DOI:** 10.25122/jml-2020-0144

**Published:** 2020

**Authors:** Alin Gabriel Sterian, Alexandru Ulici

**Affiliations:** Department of Pediatric Orthopedics, Carol Davila University of Medicine and Pharmacy, Bucharest, Romania

**Keywords:** Pediatric flatfoot, flatfoot surgery, foot deformity, minimally invasive surgery, painful flatfoot

## Abstract

Flatfoot is a common deformity in the pediatric population and has a multitude of causes. Sometimes, it can be a normal finding in children, and treatment should not be guided only based on the appearance, but rather after thoroughly assessing the patient and the impact it has on the child’s daily life. In this paper, we describe the quality of life that the patients are experiencing after the surgical treatment of this pathology. We made a comparison between the most used techniques for correcting flatfoot and insisted on the postoperative comfort of the patient, rehabilitation, and the time it took to get back to their daily routine. The comparison was made between Mosca calcaneal lengthening osteotomy, Grice extraarticular arthrodesis, arthroereisis and triple arthrodesis of the foot. All of the surgeries were performed by the same doctor at “Grigore Alexandrescu” Emergency Hospital for Children in Bucharest. From the data collected, we propose that newer, minimally invasive techniques could be used in treating this pathology in order to help the patient feel better in the postoperative period and avoid some of the complications regularly encountered when using the old techniques.

## Introduction

Foot deformities are among the most frequent reasons children are brought to the Outpatient Department (OPD) for assessment, flatfeet being the main deformity responsible for these visits [1]. From toddlers to adolescents involved in sports, the foot’s abnormal shape is a reason of concern both for parents and patients and has caused a lot of debate over the years in the academic world. Depending on the age and general considerations of the patient, it is of paramount importance to make the right diagnosis and begin a therapeutic protocol when the situation is needed [2]. Depending on multiple factors, treatment can be divided into surgical and nonsurgical, and this paper is centered on the latter. Surgical treatment is reserved for those patients where conservative treatment was initiated, but no improvement was achieved [3]. We assessed patients that underwent multiple types of surgery and tried to distinguish which one is more comfortable considering that, for our group, the results were comparable after one year. Surgery is a stressful period both for the patients and their families, so all of that sacrifice has to be done in order to improve something in the patient’s life. All of the patients enrolled in this study had their procedure done after the orthopedic treatment failed to achieve acceptable results. This included physiotherapy, orthopedic bracing, medial arch support, and changes to daily activities.

Most of the children diagnosed with flatfoot can have a normal life without any complaints. Even if the deformity is quite severe, conservative treatment is the only option if there is no functional impairment or pain. Surgical procedures are indicated when the deformity is preventing the patient from living a comfortable life. Since we are dealing with pediatric patients, sports activities are a crucial part of their lives not only for normal growth and development but also for social reasons. Hence children need to be as active as possible. Sometimes, flat feet can keep these individuals from participating in these activities, mostly because of pain, deformity, and general poor function [4]. Surgery remains the only treatment available for having painless functional feet if physiotherapy and muscle balancing exercises did not provide satisfactory results. 

**Figure 1.**
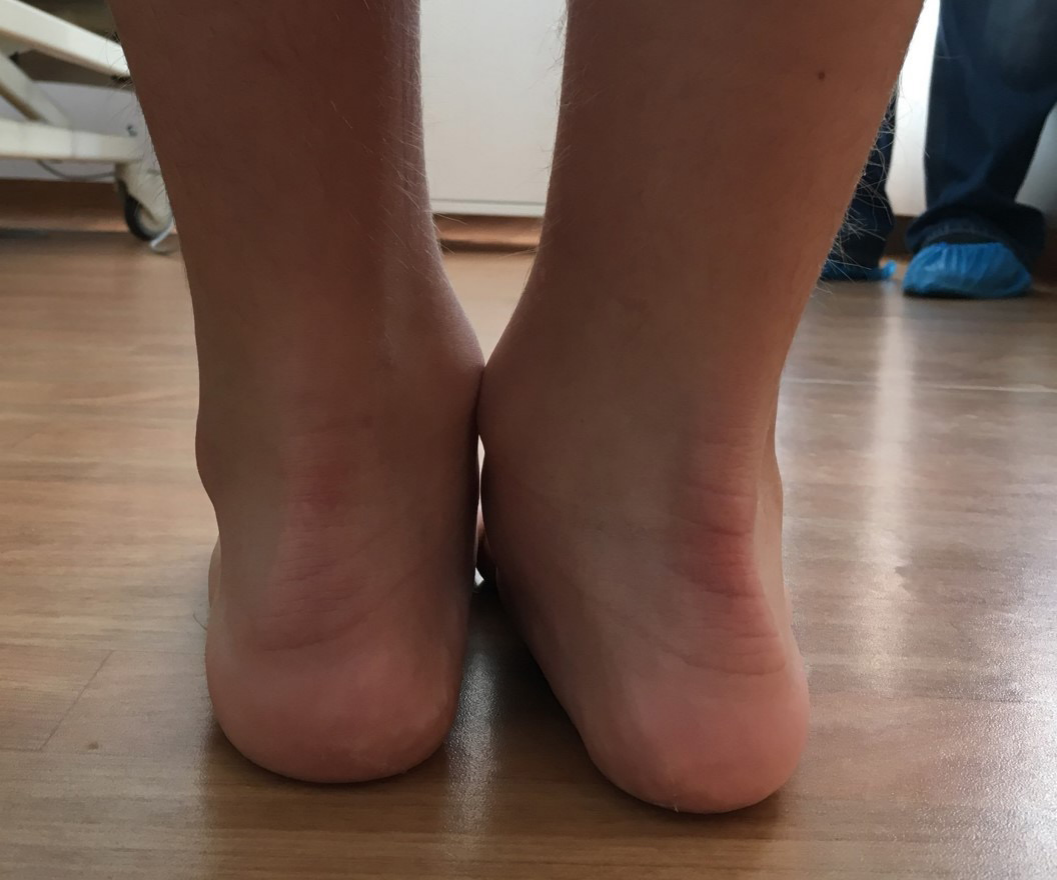
Normal (left) and pathologic (right) hindfoot valgus while full weight-bearing.

**Figure 2:**
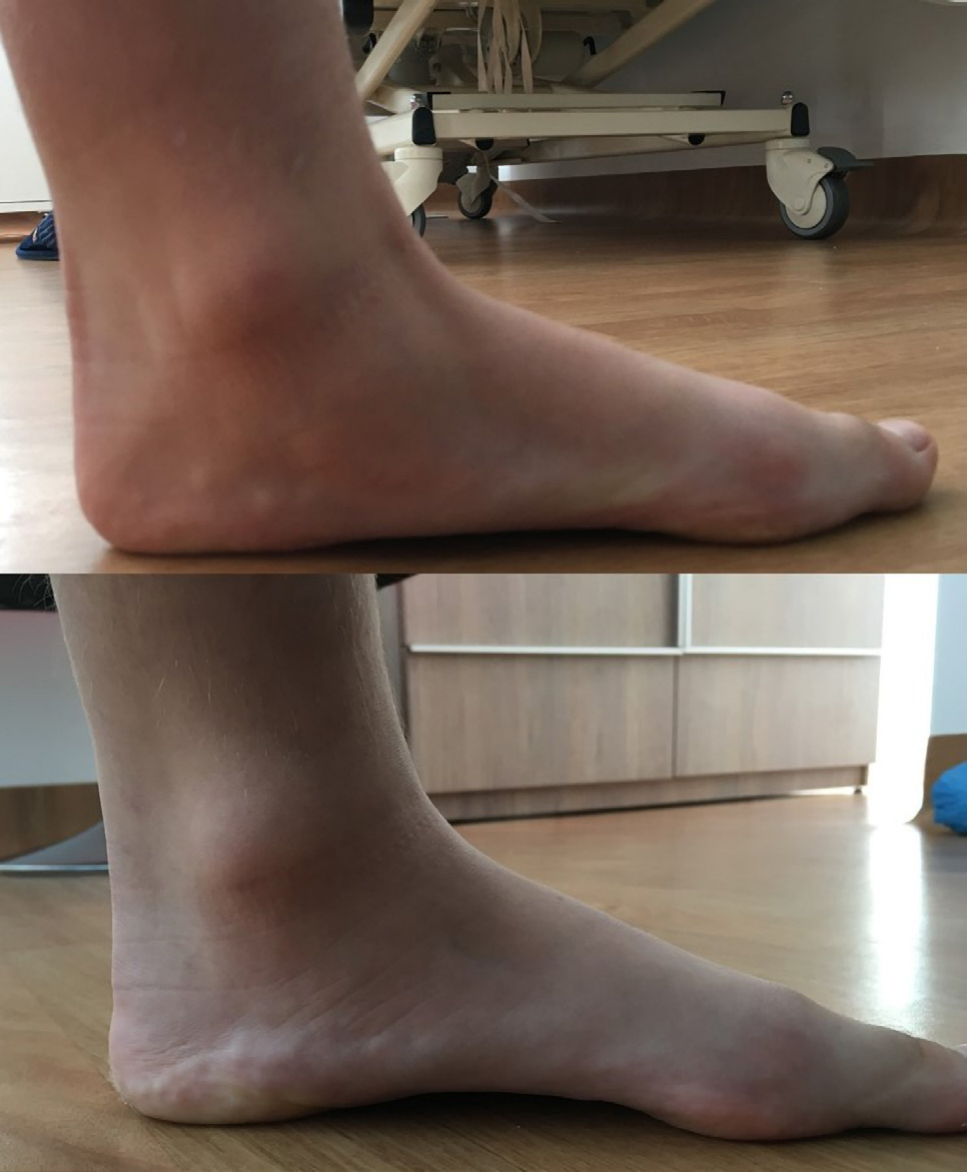
Normal (lower image) and dropped (upper image) medial arch while full weight-bearing.

The general consensus is that flat feet are diagnosed when the medial arch of the foot is dropped to a certain degree (Figures 1, 2) while weight-bearing and the in valgus alignment of the hindfoot appears because of subtalar incongruity [5, 6]. Other clinic signs for this deformity are Achilles tendon shortening, convex medial border of the foot, lateral deviation of the forefoot, and callosities on the inside of the midfoot [7]. Radiologically, full weight-bearing X-rays show an abnormal Meary angle, decreased calcaneal pitch, and increased talocalcaneal angle on the coronal view. On the frontal view, an increased talocalcaneal angle, talonavicular incongruity with a medial shift of the head of the talus that is uncovered by the navicular can be seen. Both the anteroposterior and lateral views show abnormal cyma lines [8]. Thus, although being mostly asymptomatic [9], there are many issues to take into consideration when deciding to perform surgery for these deformities. Therefore, making the right decision is crucial to achieving the desired results without putting too much stress on the patient [10, 11].

**Figure 3:**
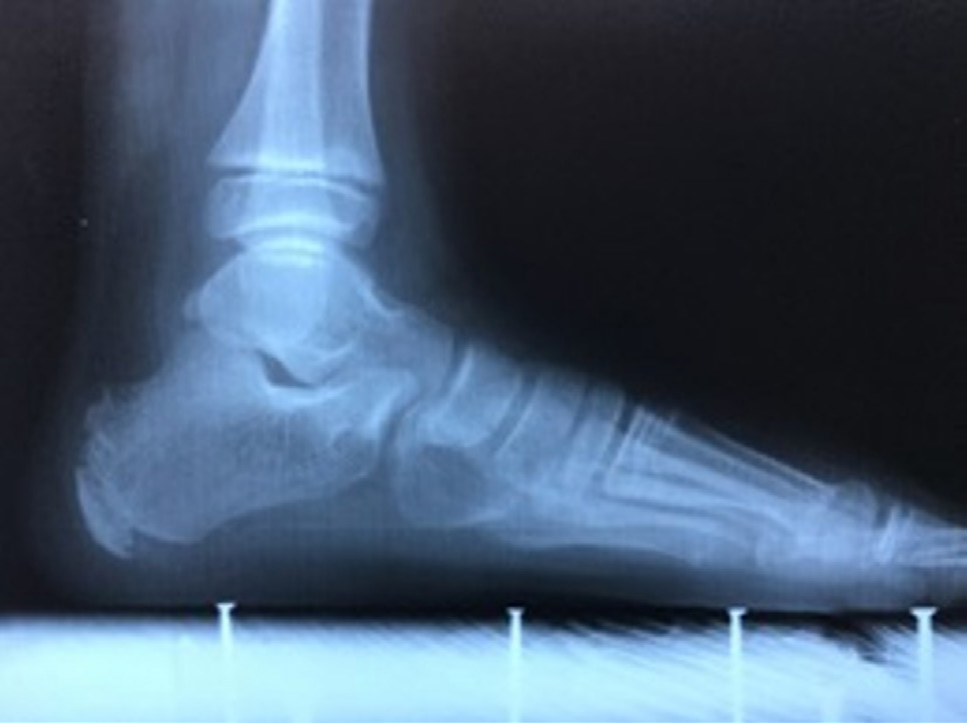
Normal preoperative full weight-bearing X-ray of the foot (lateral view).

**Figure 4:**
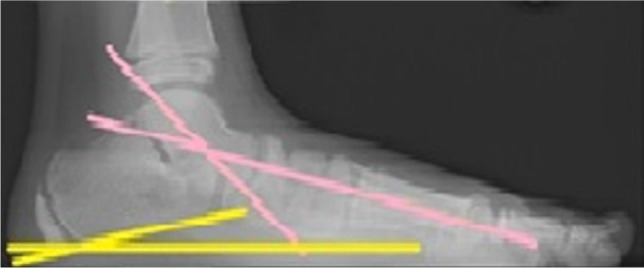
Abnormal preoperative weight-bearing X-ray of the foot. 300 Meary angle (pink) , 200 calcaneal pitch, 550 talocalcaneal angle (yellow) (lateral view).

**Figure 5:**
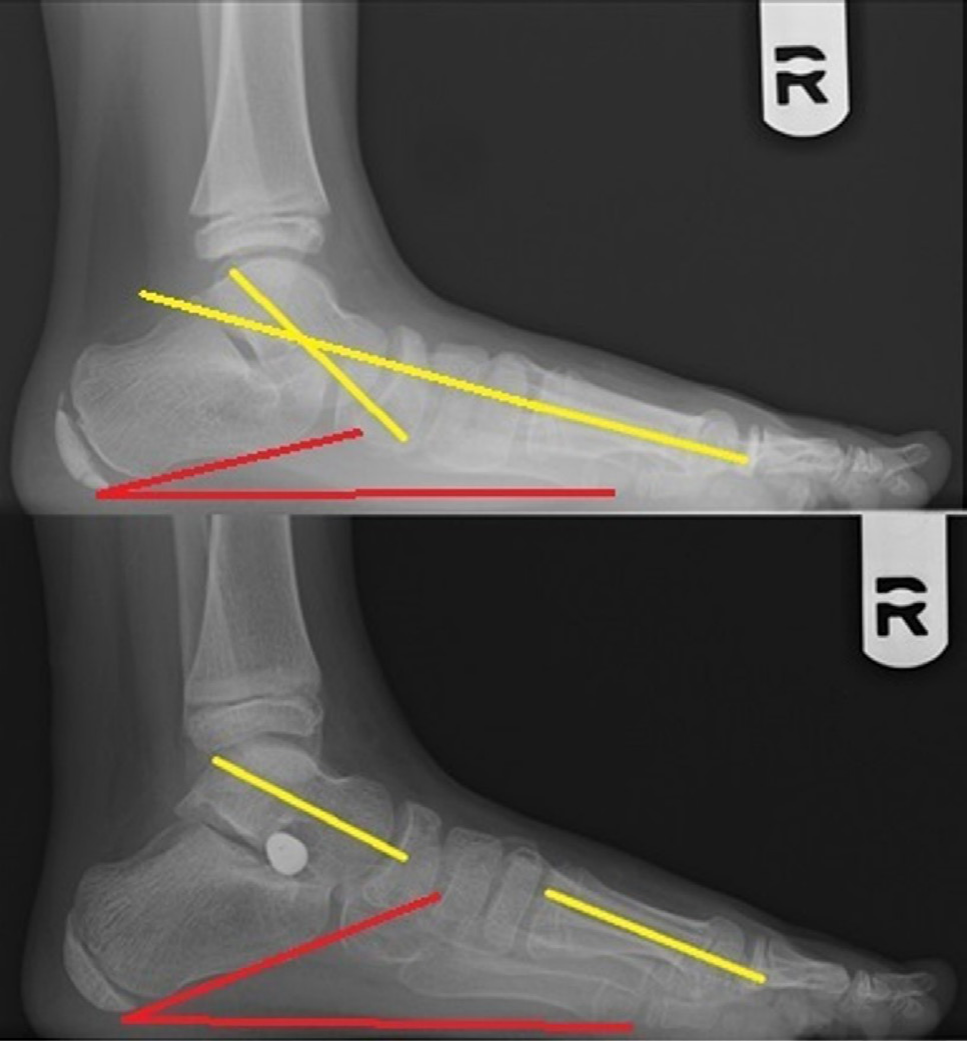
Meary angle (yellow) and calcaneal pith (red). Before (upper image) and after arthroereisis (lower image) showing good correction. Full weight-bearing lateral views.

**Figure 6:**
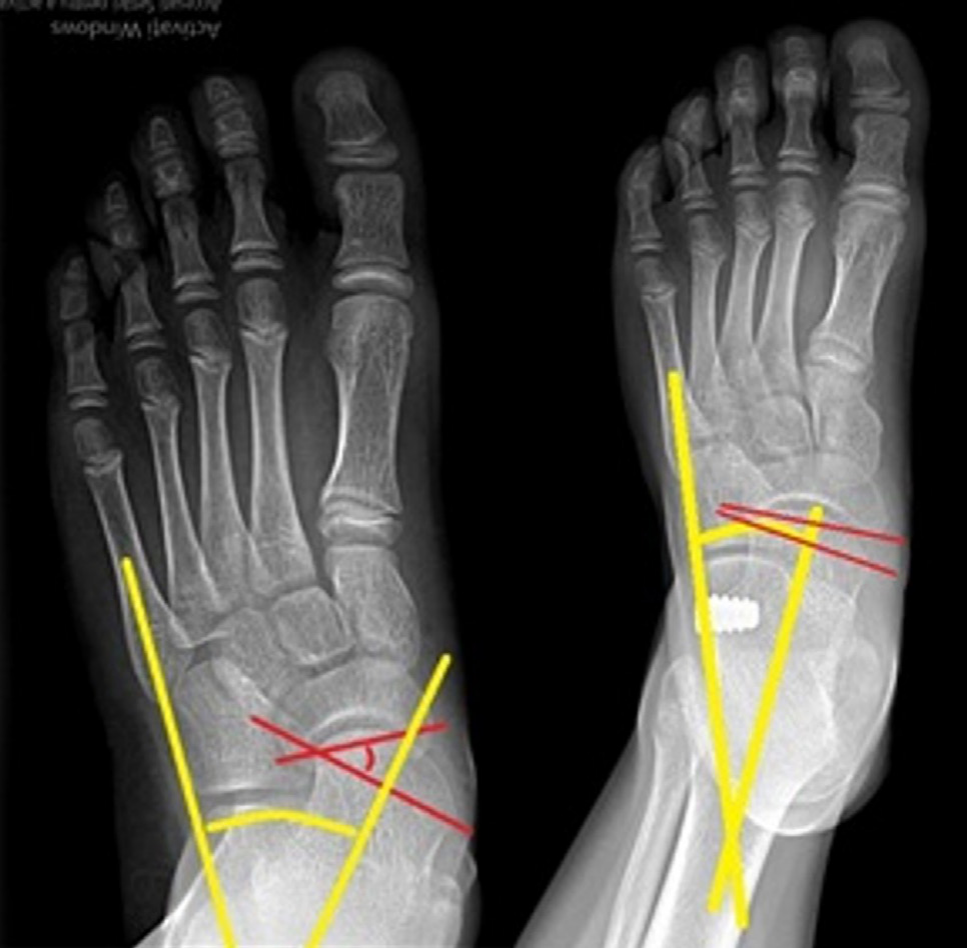
Preoperative (left) and postoperative (right) image of the same foot treated by arthroereisis. Substantial improvement of the talocalcaneal divergence angle (yellow) and navicular coverage angle (red).

**Figure 7:**
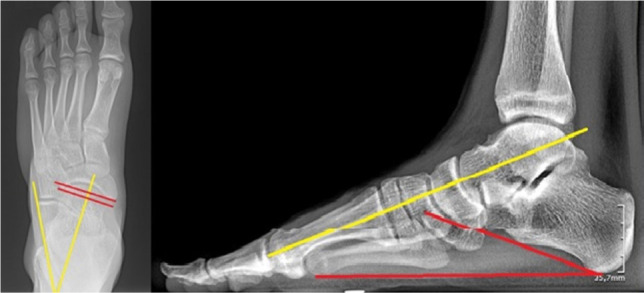
Postoperative full weight-bearing X-rays after Mosca calcaneal lenghtening. Frontal (left) and sagittal (right) weight-bearing incidences exemplifing good alignement of the bones. Frontal view: talocalcaneal divergence - yellow; navicular coverage angle - red. Lateral view: Meary angle - yellow; calcaneal pitch - red.

In our center, the most used techniques are Mosca calcaneal lengthening [12, 13], Grice subtalar extraarticular arthrodesis [14], subtalar arthroereisis [15-17] and triple arthrodesis. In this paper, it was investigated which one of these procedures has the best results and is the most comfortable for the patients without sacrificing the end result. From our experience, all of them proved to have good results, the only difference being the comfort of the patient during treatment; therefore, we could improve our protocols so that the stress of going through surgery and rehabilitation is minimal (Figures 3-7).

## Material and Methods

We assessed 39 patients that underwent a total of 70 surgeries from 2015 to 2020. The procedures listed are Mosca calcaneal lengthening, Grice extraarticular subtalar arthrodesis, triple arthrodesis of the foot, and subtalar arthroereisis. Of the 39 patients, 26 were boys and were 13 girls, with ages ranging from 9 to 17 years old. Out of these patients, 31 were diagnosed with bilateral flatfoot, 2 had unilateral right foot involvement, and 6 had unilateral left foot involvement. All of the patients diagnosed with bilateral flatfoot had the same surgical procedure done for both feet. From the 70 procedures that we performed, none was revision surgery. The procedures were divided as follows: 31 arthroereisis procedures, 21 Mosca, 16 Grice, and two triple arthrodeses. The procedures the paper is referring to are the ones performed to the feet; procedures done in conjunction with the main technique like bone graft harvesting and muscle procedures were not included. For example, we regard as one surgery when talking about the Grice procedure, and we are considering the bone harvesting and Achilles tendon lengthening surgery as a separate procedure; the same goes for Mosca when excluding iliac bone harvesting and Achilles tendon lengthening. All of our patients underwent these procedures before the foot surgery itself when choosing the Mosca or Grice protocols. These steps are mandatory for the main procedure to be carried out. Regarding complementary procedures for triple arthrodesis and arthroeresis, we have done an Achilles lengthening surgery if needed. The data collected comprised information about age, sex, unilateral or bilateral involvement, days of admission in the hospital, admission to the ward or Intensive Care Unit (ICU), unilateral or bilateral procedure, postoperative casting, associated comorbidities, postoperative complications and complains, and postoperative medical treatment. In addition to hospital and surgery topics, the study focused on the time patients need to begin normal activities, like full weight-bearing, participating in sports and social activities, days necessary for pain suppression therapy, and general comfort after the procedure.

Before the treatment plan was decided upon, we started patient evaluation with a complete clinical examination followed up by a radiologic assessment to confirm the diagnosis. The main clinical landmarks for flatfoot identified were: a drop of the medial arch, valgus deviation of the heel both plantigrade and while walking on tiptoes, modifications of medial and lateral borders of the foot, pressure sores, joint hyperlaxity and pain around the feet and legs during physical activity. All of these clinical findings were evaluated before and after surgery. The primary consideration that we centered upon was pain; it was the most important finding in deciding the treatment protocol, and it was decided to choose the surgical treatment if conservative management did not improve the patient’s condition. 

All of the patients had a collapsed medial arch, rigid hindfoot valgus, and a convex medial border. The most important clinic criteria for surgery was pain around the feet and lower legs, varying in intensity based on activity levels and severity of the deformity. Most of the patients were complaining of pain after moderate physical exercises that were keeping them from participating in normal or competitive social activities. Few patients developed callosities in critical areas that were painful after mild physical activities like short walks in the park or doing housework. 

According to the Wong-Baker pediatric pain scale [18-19], the majority of the patients were between 4 and 6, the degree of pain depending on the intensity of the physical activity they were performing. Children that were older than 12 were more involved in sports and had more painful deformities that required treatment; also, the pain was more severe in boys than girls when talking about the same level of physical activity. Table 1 shows the distribution of painful feet according to age, sex, and physical activity before and after surgery. We included in the moderate sports activity category patients that are not enrolled in supervised sports activities but are regularly active. We included patients who are doing regular sports activities and are part of a professional group of athletes who do intense training regularly in the active sports activity group.

**Table 1:** Foot pain improvement after flatfeet surgery acording to the Wong-Baker pediatric pain classification. Pain is more severe in individuals involved in profesional sports activities (defined as „active”). In some cases, there is still some residual pain after surgery but it is manageable.

Patient criteria	Sex	Age	Sports activity	Wong-Baker Pain scale preoperatively	Wong-Baker Pain scale postoperatively
**1.**	M	9	moderate	4	0
**2.**	M	17	moderate	6	1
**3.**	M	9	moderate	4	0
**4.**	M	13	active	5	0
**5.**	F	14	active	6	0
**6.**	F	10	moderate	4	0
**7.**	M	16	active	6	1
**8.**	F	12	moderate	4	0
**9.**	M	10	active	5	0
**10.**	M	10	moderate	4	0
**11.**	F	12	active	4	0
**12.**	M	15	moderate	6	0
**13.**	M	14	active	5	0
**14.**	M	9	moderate	4	0
**15.**	M	16	active	6	0
**16.**	M	10	moderate	4	0
**17.**	F	12	moderate	4	0
**18.**	F	15	moderate	5	1
**19.**	F	11	moderate	4	0
**20.**	M	12	moderate	5	0
**21.**	M	11	moderate	4	0
**22.**	M	13	active	5	0
**23.**	M	13	moderate	4	0
**24.**	M	13	moderate	4	0
**25.**	M	14	moderate	4	1
**26.**	M	16	moderate	6	1
**27.**	F	13	active	4	0
**28.**	F	12	moderate	4	0
**29.**	M	13	active	5	0
**30.**	F	9	moderate	4	0
**31.**	M	11	active	5	0
**32.**	F	14	active	6	1
**33.**	M	9	moderate	4	0
**34.**	M	10	moderate	4	0
**35.**	M	16	moderate	6	1
**36.**	M	12	moderate	4	0
**37.**	M	14	active	6	1
**38.**	M	11	moderate	4	0
**39.**	M	13	active	6	0

Radiologically, full weight-bearing X-rays of the feet in frontal and coronal views were performed. On the lateral view, the Meary angle, talocalcaneal divergence, calcaneal pitch and cyma line were evaluated. On the frontal view, the talocalcaneal angle, the talonavicular coverage angle, and the talar- first metatarsal angle were evaluated. All of the measurements were done before and after surgery in order to evaluate the degree of correction. Besides pain, that was the main issue both pre- and postoperatively, other critical factors were postoperative hospitalization days, postoperative medical treatment and the time that was required for the patients to get back to their daily routines. We evaluated the number of days that the patients stayed in the hospital postoperatively, both on the ward and in the ICU, how long was the time frame that they needed pain suppression therapy, and for long did they require antibiotic therapy.

## Results

After evaluating the 39 patients that underwent different reconstructive procedures, the general conclusion was that performing older techniques is more time consuming for the medical team and much more stressful for the patients. At the one-year follow-up, the results were comparable regarding clinical and radiologic evaluation, with no significant differences in regard to function and activity level when comparing the procedures listed. However, achieving this comfortable status was more challenging for some patients than others, and the chosen technique had a significant impact. Procedures like calcaneal lengthening and Grice implied that the patient required longer periods of hospitalization, more days of physiotherapy, a higher risk for intraoperative and postoperative complications with more inferior aesthetic results due to multiple site scarring. On the other side, a more minimally invasive technique like arthroereisis achieved the same results but implied shorter hospitalization, less pain, better comfort and aesthetic results regarding scarring. Probably the most important advantage for this procedure is that since it is atraumatic with the anatomical structures of the ankle and subtalar joint, the surgeon can perform it bilaterally, allowing the patients to avoid a second visit to the hospital and undergo another surgery.

Looking back at the data and assessing every subgroup in detail, relevant judgments were made by considering every procedure that was done. By far, the most uncomfortable procedure for the patients was the Mosca technique (concerning hospital days, postoperative treatment, days of rehabilitation, overall aesthetics, and comfort for the patient). About 5 to 7 days of hospital stay were needed for the patients to feel comfortable and safe to go back home; most of the discomfort was due to the pain associated with bone grafting (16 patients) and the calcaneal osteotomy (13 patients). Because the patients were in discomfort, physiotherapy was initiated later (5 days postoperatively until the patient was comfortable enough to mobilize. Also, the postoperative period was stressful for the family as well since bathroom activity, and general mobilization was more difficult for these patients. At 12 months postoperatively, even if the results were good, most of the patients were complaining because of scarring (10 patients) and the time they needed to get back to their normal lives (around 10 weeks for painless full weight-bearing). Also, in some cases, pain suppression therapy had unpleasant side effects like nausea, dizziness and lethargy.

The Grice procedure, on the other hand, was better tolerated by the patients, and pain management was a lot easier since the procedure itself is less invasive than others. The only problems that patients were complaining about were scarring in a very limited region (8 patients) and neurologic deficits because the peroneal nerve can get irritated during graft harvesting (4 patients). Patients were feeling better about the procedure and regained full weight-bearing faster compared to the Mosca and triple arthrodesis procedures, but much slower compared to arthroereisis. Also, hospital admission days were fewer in number thanks to better pain management and shorter postoperative care.

Arthrodesis was easier to tolerate than Mosca and Grice procedures because scarring was minimal - only arthroereisis showed better results in this regard. Postoperative pain was easily managed, and because no other procedures were associated, it was easier for the patients to recover. The standard protocol of 6 weeks non-weight bearing interval was prescribed, and rehabilitation was done without complications. All of the patients involved had neurologic disorders; therefore, other joint sparing procedures were not indicated since the risk of relapse is very high.

Looking back at the arthroereisis procedure and comparing it to the other ones, it should be said that it was the most pleasant out of the 4 for the patients to go through. One of the most important elements to consider while treating patients this way is that the procedure can be done bilaterally, avoiding further hospital admission. In general, the patients stayed about 48 hours in the hospital for the procedure compared to 5-7 days when performing unilateral Mosca, Grice, or triple arthrodesis procedures. Getting back to normal activities and painless full weight-bearing was noted about 10 days postoperatively in cases where a percutaneous Achilles tendon surgery repair was not performed, and 4 weeks if the lengthening was mandatory. Pain treatment was administered for shorter periods, and the aesthetic results were the best since scarring is minimal.

## Discussion

There are several options for treating flatfoot in symptomatic patients, and the choice for the procedure is up to the surgeon who has to consider multiple factors. The main problem with this pathology is not the aesthetic issue that the parents and, sometimes, the patients are referring to, but how the foot performs during activity, pain being the biggest complaint. The amount of pain depends mostly on an underlining pathology or type of physical activity. Professional athletes are complaining more about pain than children who occasionally participate in sports activities. Looking back at the study group, it is safe to say that doing less invasive techniques like arthroereisis can be a valid option for treating painful flatfoot. We can conclude that using this procedure for patients who do not have an underlining pathology and are involved in sports activities can consistently improve their daily lives without the drawbacks of major surgeries like Mosca and Grice. Furthermore, given the fact that Achilles tendon lengthening surgery is not mandatory, casting can be avoided until full weight-bearing is resumed. Other considerations in favor of this procedure are better aesthetic, fewer pain medications, reduced risk of feet infection, reduced infection risk if other surgeries are performed, and a much shorter rehabilitation period after surgery. One key consideration in promoting arthroereisis is that it leaves the option for the surgeon to do revision surgery without having to struggle with the sequelae of previous surgeries. It does not harm the bony structures since no bone is cut, and only the soft tissues around the subtalar joint are disrupted. In case of relapse, the procedure can be revised with a different type of implant, or the procedure can be converted to calcaneal lengthening or Grice procedure without having to deal with abnormal anatomy of the calcaneus or thallus. 

## Conclusion

Subtalar arthroereisis is a viable procedure in its own rights, but the surgeon’s decision is the driving force when treating flatfoot, especially if the patient has an underlying pathology like cerebral palsy, fibular deficiency, tarsal coalition, severe joint hyperlaxity, or other collagen- related syndromes. It is a procedure reserved for painful flatfoot when patients do not have a local or general associated pathology, and it will not produce the desired results in complex cases.

## Conflict of Interest

The authors declare that there is no conflict of interest.
